# Development of an Antifungal Edible Coating for Avocado Fruit from Avocado Residues By-Products Through a Circular Economy Approach

**DOI:** 10.3390/foods15111951

**Published:** 2026-06-01

**Authors:** Raquel Villanova-Estors, Laura Settier-Ramírez, Raquel Heras-Mozos, Gracia López-Carballo, María Bernardita Pérez-Gago, Lluís Palou, Pilar Hernández-Muñoz, Rafael Gavara

**Affiliations:** 1 Packaging Lab, Instituto de Agroquímica y Tecnología de Alimentos, IATA-CSIC, Av. Agustín Escardino 7, 46980 Paterna, Spain; raquel.villanova@iata.csic.es (R.V.-E.); r.heras@iata.csic.es (R.H.-M.); glopez@iata.csic.es (G.L.-C.); phernan@iata.csic.es (P.H.-M.); 2 Food Science, Technology and Management PhD Program, Universitat Politècnica de València, Camino de Vera, s/n, 46022 Valencia, Spain; 3 Centre de Tecnologia Postcollita (CTP), Institut Valencià d’Investigacions Agràries (IVIA), 46113 Montcada, Spain; perez_mbe@gva.es (M.B.P.-G.); palou_llu@gva.es (L.P.)

**Keywords:** avocado, *Persea americana*, anthracnose, *Colletotrichum gloeosporioides*, ethyl lauroyl arginate, active coating, postharvest quality

## Abstract

The environmental impact of food waste and agro-industrial by-products has promoted the development of circular economy strategies for food applications. In this study, edible films were developed from biopolymers extracted from avocado peel and seeds (hemicellulose, pectin, lignin, and starch), incorporating ethyl lauroyl arginate (LAE^®^) as an antifungal agent. The activity of LAE^®^ was evaluated against *Colletotrichum gloeosporioides* on inoculated avocados stored at 12 °C and 22 °C. Fruit shelf life was assessed through physiological, physicochemical and sensory parameters during cold storage and subsequent shelf life. Films containing 10% LAE^®^ exhibited strong antifungal activity, and their efficacy was higher at 12 °C than at 22 °C. Coated fruits exhibited a ripening delay of up to 2 days compared to controls. These findings highlight the potential use of avocado by-product-based LAE^®^ coatings as a sustainable strategy for preserve postharvest avocado quality.

## 1. Introduction

The massive generation of food waste represents one of the most significant environmental, social and economic challenges of the 21st century. According to the United Nations Environment Programme (UNEP), approximately 931 million metric tons of food were wasted in 2019, equivalent to around 17% of total production intended for human consumption [1]. This situation not only contributes substantially to greenhouse gas emissions but also exacerbates the inefficient use of scarce natural resources such as water, soil and energy [2]. In this context, the circular economy (CE) is presented as a transformative model capable of closing material cycles and recovering waste, especially in the food supply chain, where the potential for reducing losses is considerable [3].

Food waste in the agri-food industry is a structural problem that occurs throughout the entire supply chain, from primary production to processing, distribution, and sales. This phenomenon also has a considerable environmental impact. In addition, the decomposition of organic waste in landfills releases methane, a greenhouse gas with a global warming potential much higher than carbon dioxide. Overall, industrial food waste directly contributes to environmental degradation, inefficient use of natural resources, and climate change, reinforcing the urgent need to adopt circular models that prioritize the prevention and recovery of this waste [4,5].

In this context, one of the most vulnerable sectors in terms of food waste generation is the postharvest stage of fruit and vegetables. These products are highly perishable, and losses are intensified by pathogen activity, senescence, temperature changes, and inefficient storage and transport management [6]. A promising strategy to reduce both postharvest losses and waste is to use food waste as raw material to develop edible coatings or extract bioactive compounds with antimicrobial or antioxidant properties. For example, Salimi et al. [7] valorized apple waste through the extraction of pectin and phenolic compounds for use in edible coatings, and Settier-Ramírez et al. [8] developed edible coatings entirely made from apple residues, incorporating live antifungal yeasts to enhance their activity.

Avocados, as climacteric fruits, have a high respiration rate and intense metabolic activity, which make them particularly susceptible to postharvest deterioration, manifested by changes in external color, darkening of the flesh, loss of firmness, and reduction in organoleptic quality [9]. Strategies to improve the shelf life of avocados have received a lot of attention. Postharvest treatments include cold storage, controlled and modified atmosphere, irradiation, use of chemicals, antagonistic microorganisms, essential oils, and edible coatings, among others. Each technology has its advantages and disadvantages in avocado preservation. Among them, edible coatings have emerged as a practical, economical, and environmentally friendly option for extending the shelf life of this fruit [10]. The use of edible coatings made from polysaccharides, proteins, and lipids is a sustainable alternative for preserving avocados by forming a physical barrier that regulates moisture and gases, delaying ripening. In addition, they can incorporate active ingredients with antioxidant and antifungal properties, such as low-toxicity chemicals, essential oils or natural extracts classified as food additives or generally recognized as safe (GRAS) compounds, which can help control fungi such as *Colletotrichum gloeosporioides*, the main causal agent of anthracnose in avocados. In this sense, edible coatings stand out for their potential to replace synthetic fungicides, reduce economic losses, and align with the principles of the circular economy, as some coatings are made from agro-industrial by-products [8,11].

Ethyl lauroyl arginate (LAE^®^) is a cationic surfactant known for its powerful antimicrobial activity against bacteria, yeasts and molds. Its effectiveness is due to its action on the cell membranes of microorganisms. Furthermore, it is considered non-toxic and is therefore classified as a food additive or GRAS compound, as it rapidly breaks down in the human body into natural components. LAE^®^ has been used in a wide range of foods and has also shown great potential in antimicrobial packaging systems, improving the safety and quality of food products [12].

The present study focuses on the development of bioactive edible coatings formulated from biopolymers extracted from avocado by-products with LAE^®^ as the antifungal agent. The main novelty of this work lies in the first reported use of LAE^®^ incorporated into an edible coating to control *Colletotrichum gloeosporioides*. While previous studies have addressed the valorization of agro-industrial residues for coating production or the application of LAE^®^ in antimicrobial packaging systems, the combination of waste-derived biopolymer matrices with LAE^®^ for direct postharvest disease control in fresh fruit has not been previously reported. Moreover, this study evaluates the performance of the coatings under realistic postharvest conditions, including fungal suppression in artificially inoculated fruit and the extension of avocado shelf life during cold storage followed by a shelf-life period. By integrating circular economy principles with an effective and GRAS-classified antifungal compound, this work provides a novel and sustainable approach to simultaneously reducing postharvest losses and valorizing avocado processing by-products.

The aim of this study was to obtain bioactive coatings made from avocado by-product extracts incorporating LAE^®^ as the active compound. The films were applied to avocados previously inoculated with *Colletotrichum gloeosporioides* to assess their effectiveness in inhibiting fungal growth under post-harvest conditions. In a subsequent phase, the formulation was tested for its ability to extend the shelf life of avocados during cold storage followed by a shelf-life period.

## 2. Materials and Methods

### 2.1. Materials

Avocados (*Persea americana* Mill.) cv. ‘Hass’ were supplied by the Institute of Subtropical and Mediterranean Horticulture (IHSM) from an orchard located in Málaga, Spain. The fruits were harvested with a dry matter content of 28.7 ± 1.3%, 180 ± 20 g and an equatorial diameter of 6.6 ± 0.6 cm. No commercial postharvest treatments were previously applied.

LAE^®^ was provided by Mirenat (Vedeqsa, Barcelona, Spain). Glycerol was provided by Sigma (Barcelona, Spain).

### 2.2. Obtaining of Avocado Residue Extracts

Avocado peels and seeds were removed from ripe fruit and dried at 37 °C for 36 h. Then, the dried residues were ground separately using a Fitzpatrick comminuting mill fitted with a 500 μm screen (Model D, the Fitzpatrick Company, Chicago, IL, USA) and stored at −80 °C until use.

Residue processing was performed according to a previously described methodology by Heras-Mozos et al. [13]. To do so, dry peel was mixed with distilled water in a ratio of 1:6 and heated at 121 °C for 15 min. Then, the liquid was filtered and the solid fraction was subjected to an alkaline solution of 2% (*w*/*v*) NaOH at a ratio of 1:10 and heated at 121 °C for 15 min. The liquid extract was recovered by filtration, and the residual avocado peel was discarded. The first extract was mainly composed of a non-cellulosic polysaccharide with 3.7 ± 0.2% dry mass. Meanwhile, the second extract was mainly composed of lignin with 6.0 ± 0.3% dry mass [13].

The avocado seed was used for starch extraction through a physical method based on mechanical grinding together with the extraction solvent, in this case water. To do this, the seed powder was mixed with distilled water in a ratio of 1:20. The mixture was crushed for 3 min in a commercial blender in order to break down the fibers and release the starch granules into the aqueous medium. The mixture was then shaken vigorously for 3 h at 40 °C on a magnetic stirrer and centrifuged at 8000 rpm for 5 min. The supernatant was discarded and the pellet, containing the starch granules, was dried at 50 °C for 24 h.

### 2.3. Preparation of Coating Formulation

The polysaccharide liquor obtained from avocado peel (first extraction) was mixed with avocado starch (HS) in a ratio of 1:1 (*w*/*w*). The mixture was gelatinized by heating at 90 °C for 15 min. It was then cooled down to 50 °C with constant stirring, and glycerol (40% *w*/*w*) was gradually incorporated. Lignin (L) (second extraction) and LAE^®^ were added at 10% (*w*/*w*, based on polymer dry mass) as active compounds. This amount of LAE^®^ was selected in previous studies as the most suitable for film formulation. Lignin was directly incorporated from lignin-enriched liquid extract, considering its dry matter. LAE^®^ was incorporated into a glycerol solution with 20% of LAE^®^ (*w*/*w*), so the formulations with LAE^®^ were not supplemented with extra glycerol. Therefore, the final formulations in the study were (1) HS-L, consisting of a polymeric matrix composed of 50% avocado non-cellulosic polysaccharides and 50% starch (dry basis), supplemented with 40% glycerol and 10% lignin (*w*/*w*, based on polymer dry mass); and (2) HS-L-LAE^®^, corresponding to the same formulation and incorporating 10% LAE^®^ (*w*/*w*, based on polymer dry mass).

### 2.4. Properties of Coating Formulations

The pH of the formulations was determined using a digital pH meter (Hach Sension plus, Barcelona, Spain). The viscosity of the emulsions was measured at 25 °C using a Brookfield viscometer (Model LVF, Brookfield Engineering Laboratories, Inc., Stoughton, MA, USA). Each formulation was tested in triplicate and the results were expressed in centipoise (cp). Emulsion stability was evaluated following a modified protocol based on Valencia-Chamorro et al. [14]. A volume of 25 mL of each formulation was transferred into a volumetric flask and maintained at 25 °C for 24 h. Stability was determined by calculating the percentage of phase separation relative to the total height of the emulsion in the container. These tests were conducted in duplicate.

### 2.5. Antifungal Activity of Coating Against Postharvest Anthracnose Caused by Colletotrichum gloeosporioides

#### 2.5.1. Isolation and Identification of the Pathogen

The fungus responsible for postharvest anthracnose of avocado was isolated from a fruit showing typical anthracnose symptoms collected in the Valencia area. Spores were aseptically transferred to potato dextrose agar (PDA, Scharlab, Barcelona, Spain) Petri dishes, incubated at 25 °C in darkness, and purified through successive subcultures on PDA plates. After verifying its pathogenicity on fresh avocado fruits, the fungus was molecularly identified as *Colletotrichum gloeosporioides sensu stricto* (Penz.) Penz. & Sacc. by an external diagnostics laboratory (Laboratorio MDC, Alicante, Spain) by sequencing the DNA regions ITS, calmodulin, and β-tubulin. The strain, named *C. gloeosporioides* ACV-1, was stored in potato dextrose broth (PDB) with 20% glycerol at −80 °C and deposited in the IVIA-CTP collection of fungal postharvest pathogens. Before the experiments, the fungus was reactivated by culturing it in sterile PDA medium and incubating at 28 °C. Weekly subcultures were performed in order to have always-fresh inoculum for the inoculation trials.

#### 2.5.2. Fruit Preparation and Fungal Inoculation

Ripe avocados, in ripeness stage 3 in according to the California Hass avocado ripeness guide [15], were manually selected to ensure uniformity of size and external color, discarding those that showed decay or any type of external damage. Before use, the fruits were randomized and the surfaces disinfected by immersion in a 2% sodium hypochlorite solution for 4 min, followed by rinsing with tap water and air-drying at room temperature (22 °C). To prepare the inoculum, the spores were collected from PDA cultures and suspended in peptone water containing 0.05% (*v*/*v*) Tween 80. The spore concentration was adjusted to 10^5^ spores/mL using a Neubauer counting chamber.

Avocados were wound-inoculated by creating a single artificial lesion in two opposite areas of the equatorial region. Wounds were made using a sterile punch (0.7 mm in diameter and 5 mm in depth) previously immersed in the spore suspension. After inoculation, the fruits were allowed to air-dry for approximately 4 h at room temperature. Next, the HS-L and HS-L-LAE^®^ coatings were manually applied, simulating coating application on a fruit packinghouse roller conveyor belt. For this, 1 mL of the corresponding formulation was applied on the surface of each avocado with a micropipette and gently rubbed with gloved hands. Avocados treated with distilled water were used as control fruits. Coated fruits were then allowed to air-dry completely at room temperature before being stored. For each treatment, 18 fruits were used, of which 9 were stored at 12 °C (simulating supermarket refrigerating conditions) and the other 9 at 22 °C (room temperature), both with a relative humidity (RH) of 55 ± 5%, for 18 days. Relative humidity and temperature conditions were selected according to the specific objective of the antifungal inoculation assay. The assay was designed to evaluate the effectiveness of the coating under both refrigerated (12 °C) and room temperature (22 °C) conditions, while a moderate relative humidity (55 ± 5%) was chosen to permit controlled pathogen development while avoiding excessive surface moisture, which could promote uncontrolled fungal growth and mask the true antifungal performance of the coating.

#### 2.5.3. Assessment of Disease Incidence and Severity

The incidence and severity of anthracnose on coated fruits were evaluated after 6, 12, and 18 days of storage at both storage temperatures of 12 and 22 °C. Incidence was expressed as the percentage of infected wounds relative to the total number of inoculated wounds. Disease severity was assessed as the percentage of affected surface area showing visible anthracnose symptoms, using a categorical scale based on surface involvement (0%, 25%, 50%, 75%, and 100% of surface affected), detailed in Table S1 of the Supplementary Materials. On this scale, 0% corresponds to fruits with no visible signs of infection, while 100% indicates fungal decay completely covering the fruit surface. Results were presented as the percentage of fruits within each range of the scale. At the end of the 18-day storage period, the avocados were longitudinally sectioned, and the severity of the internal infection was evaluated by visual inspection.

### 2.6. Effect of Coatings on Avocado Quality During Storage

#### 2.6.1. Avocado Preparation

‘Hass’ avocados were stored at 5 °C and 90% RH for 1 week before use with a ripeness maturity stage of 1 [15]. Fruits were selected and the surfaces disinfected and coated with HS-L and HS-L-LAE^®^ according to the procedure described above. Distilled water was applied as control. Once dried, coated avocados were distributed in plastic boxes and stored at 5 °C and 90% RH for 2 weeks followed by 10 days of shelf life at 20 °C and 90% RH. These conditions were selected to reproduce realistic postharvest conditions commonly used to minimize dehydration. For each day of analysis, 20 fruits per treatment were used. In this case, the studies were carried out under high relative humidity conditions, simulating typical postharvest storage environments and imposing a more demanding scenario to evaluate the stability and barrier performance of the edible coating.

#### 2.6.2. Firmness

Flesh firmness of avocados was evaluated using an Instron Universal Machine (Model 3343, Instron Corp., Canton, MA, USA). A small circular section of peel, approximately 2 cm in diameter, was removed from the equatorial zone of each fruit. Firmness was determined as the maximum force required to penetrate the fruit flesh with a probe of 8 mm in diameter at a speed of 5 mm/s. For each day of analysis, 20 fruits per treatment were evaluated and the results are expressed in Newtons (N).

#### 2.6.3. Dry Matter Content

Dry matter content (DM) was determined by drying longitudinal slices of flesh (approximately 20 g) from 10 fruits per treatment in a forced-air oven at 70 °C until constant weight. The fresh- and dry-weight data were recorded and used to calculate the corresponding DM percentages.

#### 2.6.4. Weight Loss

Weight loss was assessed by individually weighing 20 avocados per treatment throughout the storage period using a calibrated analytical balance. Results were expressed as the percentage of weight loss relative to the initial weight.

#### 2.6.5. External and Internal Color

Skin and flesh color were measured using a CR-300 colorimeter (CE Minolta, Tokio, Japan) using the CIE L* a* b* color space, with a D65 light source and a 10° observer angle. For skin color, measurements were performed at two opposite points on the equatorial zone of each fruit. To evaluate flesh color, fruits were cut longitudinally, and measurements were taken immediately to minimize oxidation effects. Flesh color was measured at three specific points: two points in the equatorial zone between the skin and the seed (on either side of the seed), and one point in the apical internal region (top part of the fruit between the skin and the seed). The a* and b* values were used to calculate the hue angle (h°) using the equation h = arc tan b*/a* and chroma (C*) using the equation C = [(a*)^2^ + (b*)^2^]^1/2^. A total of 20 fruits per treatment were measured on each day of analysis.

#### 2.6.6. External and Internal Disorders

A visual evaluation of both external and internal fruit quality was performed using three replicates of ten fruits per treatment. The Hass avocado board [16] was used as a reference to identify the disorders to be evaluated. The external defects assessed included lenticel damage (characterized by black, collapsed lenticels), chilling injury (manifested as sunken black lesions), and potential phytotoxic effects associated with coating application, indicated by the development of peel alterations (black spots, peel discoloration, uneven ripening). Internal defects assessed included flesh adhesion to the seed (caused by uneven ripening) and flesh browning (characterized by bruising and grey pulp). The severity of each defect was rated using a scale from 1 to 5 based on the percentage of the surface area affected, following methodologies previously reported for Hass avocado [17,18,19]: 1 = no defect; 2 = <25% of surface area affected; 3 = 25–50% of surface area affected; 4 = 50–75% of surface area affected; 5 = >75% of surface area affected.

#### 2.6.7. Respiration Rate and Ethylene Production

CO_2_ and ethylene production were measured on 4 replicates per treatment. Each replicate comprised two fruits placed in sealed containers of known volume. The accumulation of CO_2_ and ethylene in the jar was measured at 20 °C after 2 h. One mL of the headspace was then injected into a Perkin-Elmer Model 2000 gas chromatograph (Norwalk, CT, USA) equipped with a Poropak QS 80/100 column, a thermal conductivity detector for CO_2_, and a flame ionization detector for ethylene. Helium served as the carrier gas at a pressure of 63.4 kPa and 55.2 kPa for CO_2_ and ethylene analysis, respectively. The temperatures for the injector, oven, and detector were set at 115 °C, 35 °C, and 150 °C, respectively, for CO_2_ measurement. For ethylene analysis, the injector, oven, and detector temperatures were 175, 75, and 175 °C, respectively. The respiration rate of the fruits was expressed as mL CO_2_·kg^−1^h^−1^, while ethylene production was expressed as μL C_2_H_4_·kg^−1^h^−1^.

#### 2.6.8. Sensory Analysis

To evaluate how coatings affected avocado quality over time, a sensory test was conducted with 13 semi-trained panelists. Control samples and those coated with HS-L and HS-L-LAE^®^ were coded with a three-digit numerical code. Following the attributes described by Giuggioli et al. [9] with slight modifications, the brightness of the whole fruit was evaluated, and for avocado pulp the color intensity, aroma intensity, sweetness, bitterness, flavor intensity, creaminess and firmness were evaluated. Quality attributes were evaluated using a 9-point hedonic scale, where 0 represented little or no intensity and 9 represented high intensity. The study was conducted in a standardized room in accordance with ISO 8589 2010 [20].

### 2.7. Statistical Analysis

Statistical analysis was performed using a one-way ANOVA with SPSS software vs 26.0 (SPSS Inc., Chicago, IL, USA). Values were expressed as means ± SE. Significant differences among means were determined using Fisher’s protected LSD test (*p* < 0.05). For disease incidence data presented in Section 3.2, error bars were calculated as binomial standard error based on the total number of fruits analyzed per treatment.

## 3. Results

### 3.1. Properties of Coating Formulations

Viscosity is a key parameter affecting the homogeneity, thickness and continuity of coatings, as well as their potential for industrial application. In this study, the viscosity of HS-L formulation was 11.3 ± 0.5 cP, while the addition of LAE^®^ increased the viscosity to 18.6 ± 0.6 cP. The relatively low viscosities, as obtained in this study, are considered optimal for postharvest industrial applications [21]. The observed increase in viscosity can be attributed to structural modifications in the coating matrix caused by LAE^®^. These changes could have improved the interactions between biopolymers (hemicelluloses, pectins, starch and lignin), favoring the formation of a denser and more stable network. Studies on the incorporation of antimicrobial additives and surfactants such as LAE^®^ have demonstrated a moderate increase in viscosity, which is attributed to electrostatic and hydrophobic interactions [10,22].

The pH of the HS-L coating was 5.30 ± 0.07, and the addition of LAE^®^ increased the pH to 6.33 ± 0.03. This increase in pH can be explained by the chemical nature of LAE^®^. It is a cationic salt derived from arginine, a basic amino acid. Its structure features a guanidinium group with a high pKa (~10–11), which acts as a proton acceptor in slightly acidic or neutral environments. When LAE^®^ is added to the coating matrix, these basic groups can accept free protons from the solution. This shifts the acid–base balance towards the formation of less acidic species, consequently raising the pH of the mixture [10,23].

On the other hand, although both formulations exhibited a 64% phase separation during the stability study, a qualitative difference was observed. In HS-L formulation, the upper phase was significantly darker and more defined than the HS-L-LAE^®^ formulation, which showed a much more subtle separation, with the upper phase being barely perceptible. This improved visual homogeneity could be due to the emulsifying properties of LAE^®^, which likely contributed to a more stable dispersion of the components and reduced the contrast between the phases. By reducing interfacial tension, LAE^®^ can minimize phase differences and delay or suppress visible separation. Similar studies have shown that adding surfactants or cationic antimicrobial agents such as chitosan improved the stability of emulsions and coatings. These agents contribute to improving system uniformity, reducing phase separation, and facilitating redispersion by manual agitation, thereby enhancing the product’s usability and visual appearance during storage [24,25]. The complete redispersion of both formulations by manual agitation indicates favorable redispersibility, which is advantageous in industrial applications. This property ensures that the formulation can be readily rehomogenized without compromising functionality, supporting consistent performance during storage and use.

### 3.2. Ability of the Coatings to Control Avocado Anthracnose

**Figure 1:**
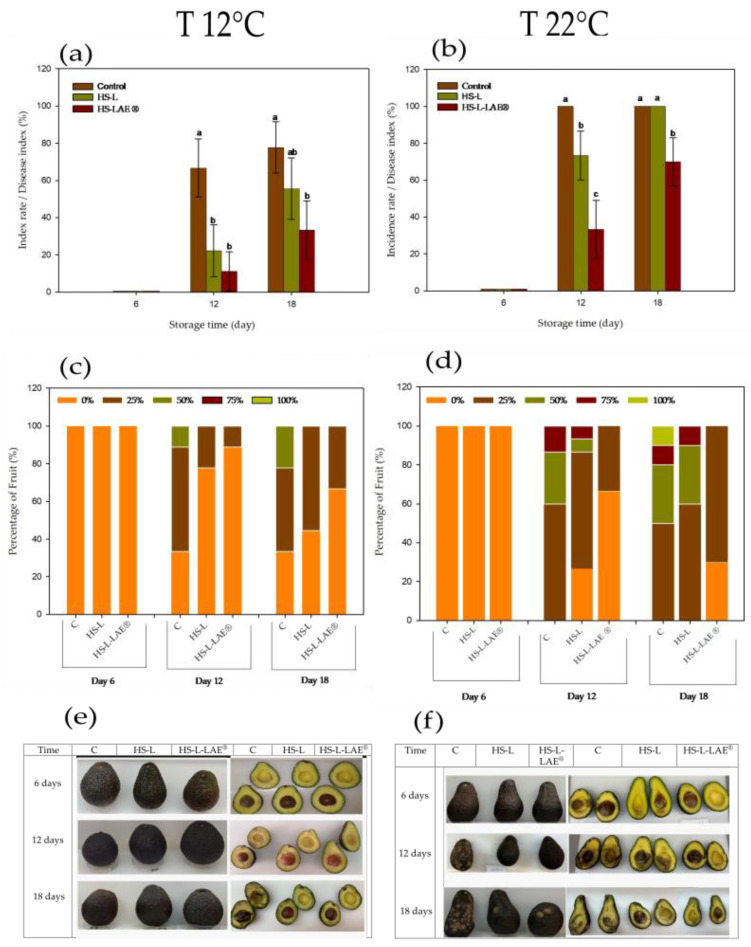
Incidence of anthracnose (%) in infected avocados stored with several coating treatments; uncoated (control) and coated with avocado residue extracts (HS-L) and ethyl lauroyl arginate (HS-L-LAE^®^) at (**a**) 12 °C and (**b**) 22 °C for 18 days (%). Distribution of uncoated and coated avocado fruits among different anthracnose damage severity levels, expressed as the surface area affected by the disease, after storage at (**c**) 12 °C and (**d**) 22 °C for 18 days. Visual appearance of the external and internal defects of the uncoated and coated avocados studied after storage at (**e**) 12 °C and (**f**) 22 °C. Error bars correspond to the binomial standard error of disease incidence percentages calculated from the total number of analyzed fruits. Statistical differences between treatments were assessed using Fisher’s protected LSD test (*p* < 0.05), different lowercase letters in subfigures (**a**,**b**) indicate significant differences between samples.

To evaluate the efficacy of the active formulations for controlling anthracnose caused by *Colletotrichum gloeosporioides*, in vivo trials were conducted at two storage temperatures (12 and 22 °C). The fruits were inoculated before applying the coatings (HS-L and HS-L-LAE^®^) to evaluate their curative activity compared to the uncoated control. The evolution of anthracnose incidence and severity is shown in Figure 1.

After 6 days of storage, no fungal growth was observed on coated or control fruits at either storage temperature. After 18 days, the percentage of infected wounds was lower on avocados treated with the active coating (HS-L-LAE^®^) compared with the control, with disease incidence reductions of 44% and 30% relative to the control at 12 °C and 22 °C, respectively. Regarding disease severity, the active coating managed to limit fungal growth to a maximum of 25% of the fruit surface, while the control reached much higher levels of infection. In general, a higher disease incidence and severity was observed on avocados stored at 22 °C, as higher temperatures favor fungal growth. Despite this, the active formulation provided protection against decay caused by *C. gloeosporioides* in coated avocados, although to a lesser extent than on fruits stored under refrigeration.

Various studies in the literature have demonstrated the effect of edible coatings formulated with chitosan and other biopolymers containing active ingredients such as essential oils, natural extracts, or biocontrol agents on the control of anthracnose in avocado [11]. For example, Obianom et al. [26] reported a 30% reduction in disease incidence in inoculated avocado fruits coated with a chitosan solution after 14 days of storage at 7.5 °C followed by 5 days at 18 °C, a result comparable to that obtained in the present study. However, to our knowledge, the effect of LAE^®^ as an antifungal agent has not been previously reported for the control of *C. gloeosporioides* in avocados. In other fruits, LAE^®^ inhibited the in vitro growth of *Penicillium expansum* and enhanced disease resistance in pears [27], and a chitosan coating containing LAE^®^ controlled decay in table grapes compared to the control fruit during 15 days of storage at 4 °C, while approximately 25% of the fruits of the control group showed signs of infection [28].

The incorporation of LAE^®^ at 10% (*w*/*w*, based on polymer content) falls within the concentration range commonly reported for antimicrobial agents embedded in edible coatings and active packaging systems. When antimicrobial compounds are incorporated into polymeric matrices, a fraction of the active substance may interact with the polymer chains through electrostatic or hydrophobic interactions, reducing the amount of freely available compound responsible for microbial inhibition [29,30]. In the case of LAE^®^, a cationic surfactant, its interaction with biopolymer matrices may partially limit its immediate bioavailability, which explains why higher nominal concentrations are often required compared to direct in vitro applications. Therefore, the concentration used in this study should not be interpreted as evidence of low intrinsic antifungal efficacy, but rather as a necessary level to ensure effective activity under in vivo conditions, where matrix interactions, diffusion constraints, and the artificially high fungal inoculum represent a more stringent challenge scenario.

### 3.3. Impact of Coatings on Avocado Quality During Storage

#### 3.3.1. Firmness

The firmness of coated and uncoated avocados throughout storage is presented in Table 1. Firmness at harvest was 230 ± 20 N, and after 2 weeks of storage at 5 °C it was reduced by 30% in coated and uncoated avocados. A sharp decrease in firmness was observed after transferring the fruit to 20 °C in all treatments. However, coated fruits maintained higher firmness values than control avocados, with significant differences observed from day 4 at 20 °C. This effect could be attributed to the ability of the polysaccharides present in the coating formulations to form a semipermeable barrier to gases around the fruit, reducing respiration rate and the activity of cell-wall-degrading enzymes such as pectin methylesterase and polygalacturonase, which are responsible for cell wall disassembly and fruit softening [31]. This trend is supported by a previous study in which oxygen permeability of HS-L and HS-L-LAE^®^ films was evaluated, confirming their potential to limit gas exchange. Additionally, the presence of lignin and starch has been reported to enhance the structural integrity of coatings, reinforcing their physical and functional protection against firmness loss [32,33]. Our results also show that the incorporation of LAE^®^ into the HS-L coating slightly enhanced this effect. In addition to its antimicrobial activity, LAE^®^, as a cationic surfactant, can electrostatically interact with anionic polysaccharides present in the coating matrix (such as pectins and hemicelluloses), promoting the formation of a more compact and cohesive polymeric network. This results in a coating with improved structural integrity and barrier capacity, which may limit gas exchange and thereby slow down firmness loss in the fruit [34]. Furthermore, the increase in elasticity of the films and the viscosity of the formulation (see Section 3.1) due to the presence of LAE^®^ can also contribute to the physical protection of the fruit, helping to preserve firmness during storage. Other studies have also demonstrated that the incorporation of cationic surfactants into polymeric matrices reinforces the coating structure and improves its barrier functionality.

#### 3.3.2. Dry Matter Content (DMC)

DMC is a critical quality parameter in avocado, as it is closely associated with flavor development, texture, and overall consumer acceptability. International standards [35] require a minimum DMC of 21–23% for the export and commercialization of ‘Hass’ avocado. Nevertheless, optimal commercial maturity, associated with improved flavor, texture, and lipid content, is typically achieved at DMC values of 26–30% [36], as was the case for the fruit selected in this study.

DMC showed no significant differences among treatments (control, HS-L, and HS-L-LAE^®^) in any of the storage periods evaluated (storage at 5 °C followed by up to 10 days at 20 °C) (Table 1). The main variation in DMC occurs during on-tree maturation rather than during postharvest storage. In general, minor changes in DMC during storage are typically attributed to water loss. However, these changes are generally minimal and not statistically significant unless dehydration is severe [37]. Consistent with the presented results, most studies report that DMC in ‘Hass’ avocado remains relatively stable during postharvest storage, particularly under short- to medium-term storage and controlled temperature and humidity conditions [18,37].

#### 3.3.3. Weight Loss

Weight loss results are presented in Table 1. No significant differences were observed among treatments at any sampling point during storage. This result indicates that the evaluated coatings provide a low barrier to water vapor, allowing transpiration rates similar to uncoated fruits. Polysaccharide-based formulations, such as those used in this study (rich in hemicelluloses, pectins, starch, and lignin), are generally characterized by limited effectiveness in reducing water loss. This behavior is characteristic of hydrophilic matrices, where water acts as a plasticizer, increasing molecular mobility and permeability [38,39]. It is especially evident when compared to coatings containing lipids, waxes, or essential oils, which enhance water vapor barrier properties due to their hydrophobic nature [40]. In a previous study, the water vapor permeability (WVP) of HS-L and HS-L-LAE^®^ films was evaluated at 75% RH, showing values in the range of 10^14^ kg/(m·s·Pa) and 10^15^ kg/(m·s·Pa), respectively. These values are considered low to moderate for HS-L and moderate for HS-L-LAE^®^. The amphiphilic properties of LAE^®^, which contains both polar and non-polar segments, allows it to integrate into the polymer matrix, altering its internal configuration and decreasing its water affinity [23]. Despite these values, no significant differences were observed between both treatments when applied as coatings on avocado fruit. This may be attributed to the high RH level (90–95%) during storage, which contrasts with the 75% RH used in the previous film study. At elevated humidity levels, increased water absorption by the coating can create hydrophilic pathways that facilitate vapor diffusion, thereby diminishing the barrier effect of LAE^®^. Another relevant aspect to consider is the possible discontinuity or lack of homogeneity in the fruit coverage since coating continuity and structural integrity are critical factors for maximizing barrier performance. The formation of microcracks, pores, or uneven distribution of the coating, due to the roughness of the avocado skin, may further increase water vapor permeability and explain the absence of significant differences compared to the control.

#### 3.3.4. External and Internal Color

Skin color is one of the main visual indicators of ripeness in Hass avocados. According to the data obtained in this work, the external color parameters L*, C* and hue significantly decreased with increasing storage, with differences between coated and control fruits at several sampling points (Table 2). After 2 weeks at 5 °C, the HS-L and HS-L-LAE^®^ coatings caused significant changes in the external color of ‘Hass’ avocados compared to the control. In particular, avocado fruit coated with HS-L-LAE^®^ had a lower L* value (31.9 ± 0.6) than control fruit (35 ± 3). Similarly, hue values were significantly lower in coated (HS-L and HS-L-LAE^®^) than in control samples, indicating a transition to less greenish and more reddish or brownish colors. This change can be attributed to optical effects generated by the presence of the coating on the fruit surface. No significant differences were detected in chroma (C*), suggesting that color saturation remained stable. It should be noted that, although the differences observed were significant, their magnitude was relatively low, so the visual impact on the consumer could be limited. These results are consistent with a previous study in which films were developed from the same formulations, showing a tendency towards brown tones and low lightness. These differences due to the coating were no longer noticeable when the color of the skin changed from green to black.

As ripening progressed at 20 °C, L* and hue values progressively decreased in all treatments, reflecting the typical darkening of the fruit and a shift from greenish tones to reddish-purple, respectively. However, coated fruits maintained significantly higher L* values than control fruits throughout storage, while the hue values of coated avocados remained higher than control fruit until day 4 at 20 °C, suggesting a delay in ripening. These results support the hypothesis that the polysaccharides, pectins, starch, and lignin present in the HS-L and HS-L-LAE^®^ coatings, along with LAE^®^, may contribute to delaying peel darkening. This effect is likely due to modulation of the respiration rate and enzymatic activity associated with chlorophyll degradation and anthocyanin accumulation, key pigments responsible for reddish and purple tones [41].

In the case of avocado pulp, no significant differences were observed between coated and uncoated avocados in L* and hue values throughout storage, indicating that neither the HS-L coating nor the HS-L-LAE^®^ coating had a relevant effect on these parameters (Table 2). This behavior aligns with studies by Garcia and Davidov-Pardo [11], who showed that avocado flesh may be less susceptible to changes in L* and hue under controlled storage conditions, especially when there is no mechanical damage, chilling injury or severe oxidative stress.

**Figure 2:**
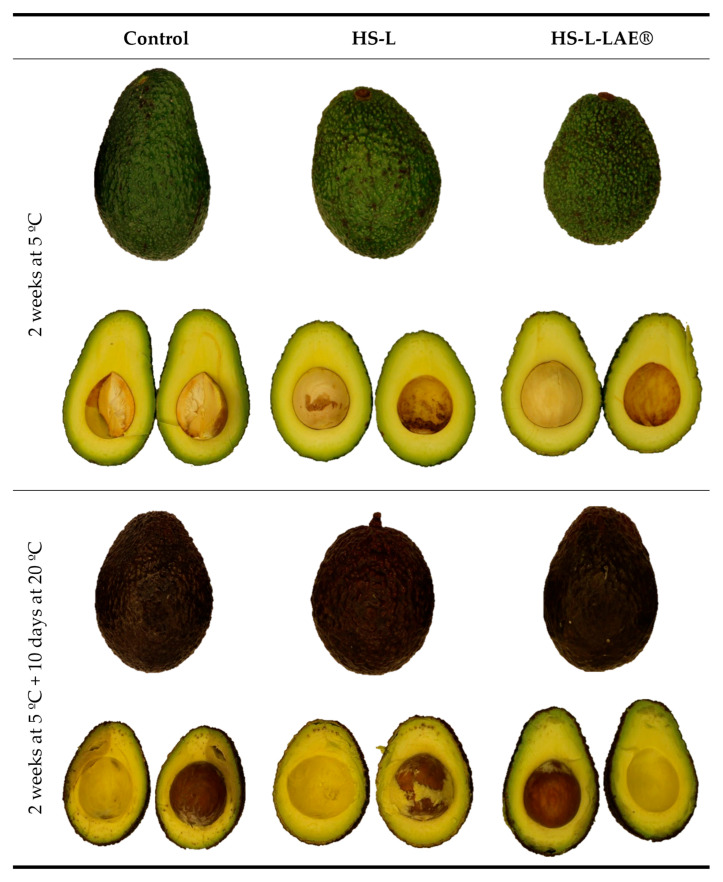
External and internal visual aspect of uncoated (control) ‘Hass’ avocados and avocados coated with HS-L or HS-L-LAE^®^ after 2 weeks at 5 °C and 2 weeks at 5 °C followed by 10 days at 20 °C.

In the case of C*, although significant differences between treatments were detected on some days of analysis, the magnitude of these differences was low; therefore, it is unlikely that these variations have a relevant visual impact on the final consumer, as confirmed in the sensory analysis (see Section 3.3.7). Overall, the results suggest that the coatings evaluated do not significantly alter the internal color of avocado pulp during postharvest, which is beneficial for maintaining the visual quality of the fruit. Furthermore, internal color changes in avocado are mainly associated with enzymatic browning caused by polyphenol oxidase (PPO), which catalyzes the oxidation of phenolic compounds into quinones that subsequently polymerize into brown pigments. Therefore, the stability of internal L*, hue and C* values observed during storage may suggest limited oxidation processes and reduced PPO-related browning in the avocado pulp. However, as shown in Figure 2 and in the evaluation of internal and external disorders, a higher incidence of fungal decay was observed in control samples. This suggests that disorder development during storage may occur in a localized manner without substantially altering global chromatic parameters. Therefore, color measurements alone may not be sufficiently sensitive to discriminate early or moderate deterioration under natural shelf-life conditions. This result is consistent with other studies in which edible coatings enriched with antimicrobial agents have demonstrated effectiveness in preserving the internal appearance of fruits during postharvest, including avocados [11,42,43] and other tropical species [44].

#### 3.3.5. External and Internal Disorders

External and internal disorders were evaluated during storage. Figure 2 shows a sample of the external and internal appearance of avocados after 2 weeks at 5 °C and after 2 weeks at 5 °C followed by 10 days at 20 °C. No external disorders, such as phytotoxicity, lenticel damage, or visible symptoms of chilling injury, were observed in any of the samples evaluated. This indicates that coatings with and without LAE^®^ did not negatively affect the integrity of the fruit epidermis or its tolerance to cold storage conditions. These findings are consistent with previous studies reporting the safety of polysaccharide-based coatings for fruit preservation [45], as well as quality maintenance of fruits and vegetables such as cantaloupe melon, lettuce, and green peppers in contact with LAE^®^ [22].

Flesh adhesion to the seed, caused by uneven ripening [46], showed high values in all treatments at harvest and after cold storage. After fruit storage at 20 °C, flesh adhesion values decreased significantly, with a faster reduction in control fruit (1.4) after 2 days at 20 °C compared to coated fruit (HS-L: 2.07; HS-L-LAE^®^: 2.53). However, at intermediate and final stages of storage (7 and 10 days at 20 °C), the differences among treatments decreased and were no longer statistically significant. This may be related to the effect of the coatings on fruit ripening and firmness loss, as polysaccharide-based coatings have been reported to delay ripening and firmness degradation by reducing transpiration and respiration rates [45].

In the case of flesh browning (flesh bruising and grey pulp), all treatments exhibited minimal values at harvest and after cold storage, indicating the absence of damage. However, during ambient storage, a progressive increase in incidence and severity was observed, particularly in the uncoated control, which reached a mean value of 2.4 at 10 days at 20 °C. In contrast, fruits coated with HS-L and HS-L-LAE^®^ showed slightly lower values (1.8 and 2.2, respectively). Internal browning in avocado flesh is primarily associated with enzymatic oxidation of phenolic compounds, mediated by PPO and peroxidase, a process that intensifies during ripening and under oxidative stress conditions [11]. Additionally, natural variability among fruits and other factors, such as mechanical damage during harvest, handling or transport, chilling injury, or preharvest conditions, can influence the severity of this disorder [37,47,48].

Nevertheless, in the present study, the observed browning was unlikely to be related to chilling injury, as the storage conditions used did not favor the development of this physiological disorder. On the other hand, although edible coatings can reduce browning incidence by limiting oxygen diffusion and creating a modified atmosphere, their ability to protect against impact-induced browning is limited, as they do not prevent initial physical damage. Although the effectiveness of the developed coatings in reducing internal physiological disorders was limited, other authors have reported that polysaccharide-based coatings and/or those containing antimicrobial agents can reduce the severity of internal browning in avocado and other climacteric fruits during postharvest storage [49,50]. It has also been noted that the effectiveness of coatings depends on their composition, thickness, and ability to maintain an optimal internal atmosphere [44].

#### 3.3.6. Respiration Rate and Ethylene Production

**Figure 3:**
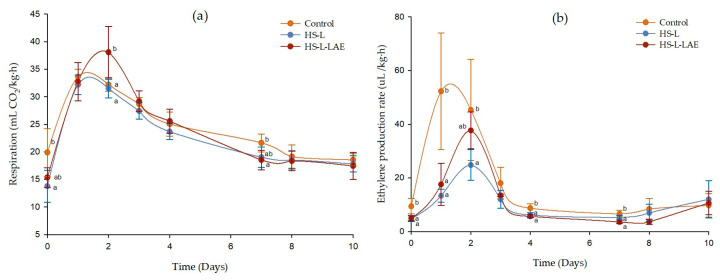
Respiration rate (**a**) and ethylene production (**b**) of uncoated (control) ‘Hass’ avocados and avocados coated with HS-L or HS-L-LAE^®^ held at 20 °C after 2 weeks of storage at 5 °C. For each storage period, different letters indicate significant differences using Fisher’s protected LSD test (*p* < 0.05). Bars represent SD.

Respiration rate (expressed in mL CO_2_/kg-h) and ethylene production of coated and uncoated avocados, as shown in Figure 3, exhibited profiles that responded to the typical climacteric pattern of this fruit, although they were modulated by the application of the coatings. After 2 weeks of cold storage, coated fruits had lower respiration than uncoated samples, with values of 15.3, 13.7 and 19.8 mL CO_2_/kg-h for HS-L-LAE^®^, HS-L, and the control, respectively. Similarly, ethylene production was lower for coated fruit (4.9 μL C_2_H_4_/kg·h for HS-L-LAE^®^ and 4.7 μL C_2_H_4_/kg·h for HS-L) than in the uncoated control (9.4 μL C_2_H_4_/kg·h). These results indicate partial inhibition of ripening metabolism caused by the modified atmosphere generated by the coatings.

During storage at 20 °C, distinct climacteric peaks of both CO_2_ and ethylene were observed. The control group reached CO_2_ and ethylene maximums after 1 day of storage at 20 °C, with 34 mL CO_2_/kg·h and 54 μL C_2_H_4_/kg·h, respectively. The observed synchrony provides clear evidence of the well-documented interaction between both processes, in which ethylene functions as a signaling hormone that triggers respiratory pathways, ultimately boosting its own synthesis [51,52]. Although the HS-L coating did not delay the CO_2_ peak compared to the control, it did delay the ethylene peak by 1 day and significantly reduced the value of both (33 mL CO_2_/kg·h and 25 μL C_2_H_4_/kg·h). The lower CO_2_ and C_2_H_4_ values in HS-L-coated fruits suggest a slowdown in ripening metabolism, which may be associated with the gas barrier effect of the coating [53]. The restriction in gas exchange between the fruit and the surrounding atmosphere delays the activity of enzymes such as ACC oxidase and ACC synthase, responsible for ethylene biosynthesis, as well as other enzymes responsible for ripening [54]. This delay in ripening is consistent with the observations seen in Section 3.3.1, where fruit firmness loss was delayed in coated avocados compared to the control group.

The HS-L-LAE^®^ coating was the only treatment that was able to delay the respiration peak by up to two days, reaching the highest value in the test (38 mL of CO_2_/kg_3_-h). Similarly, the ethylene peak was delayed by two days, with a magnitude intermediate between the control and the HS-L coating (38 μL of C_2_H_4_/kg_3_/h). This behavior suggests a moderate and more sustained hypoxic environment, induced by the greater gas barrier capacity of the LAE^®^-enriched coating, as reported for standalone films with similar composition [55]. The observed effect may be partly due to the presence of LAE^®^ in the coating, since it has been reported to alter the activities of enzymes involved in antioxidant and defense processes in vegetal tissues [56,57], which may influence the respiratory response to stress and the transition between anaerobic and aerobic metabolism.

Following the climacteric peak, all treatments exhibited a gradual decline in CO_2_ and ethylene levels. Overall, the control group presented slightly higher values than the coated fruit, reflecting greater metabolic activity that can result in faster ripening, shorter shelf life, and a possible loss of organoleptic quality [58]. Therefore, these results highlight the effectiveness of the coatings in modulating fruit physiology and extending postharvest quality.

#### 3.3.7. Sensory Quality

Sensory evaluation is crucial in the development of edible coatings, as coatings can change the organoleptic properties of the fruit [59]. The sensory analysis results shown in Table 3 indicate that edible coatings derived from avocado waste, both with and without LAE^®^, had a significant impact compared to control fruits on several sensory attributes during the first few days of storage at 20 °C following the two-week period at 5 °C. Generally, avocados treated with HS-L-LAE^®^ had higher firmness and bitterness values, and lower external brightness, external color, color intensity of flesh, sweetness and taste intensity values than the control after 2 days of shelf life at 20 °C. The increased bitterness and firmness are likely associated with delayed ripening. This interpretation is supported by the reduced ethylene production and slower firmness loss observed in HS-L-LAE^®^-treated avocados after the same storage duration, indicating a slower ripening metabolism. As ripening is typically accompanied by the degradation of or a reduction in bitter-related compounds such as tannins, delayed maturation may explain the persistence of these compounds and the higher bitterness scores observed in coated fruits [52]. Sweetness, creaminess and firmness values for the HS-L coating were intermediate between the control and HS-L-LAE^®^. After 4 days of storage at 20 °C, significant differences were still observed between the HS-L-LAE^®^ and the other treatments in terms of external brightness and color, color intensity, and flavor intensity. From day 7 onward, no significant differences were generally observed between treatments for any attribute. These results are consistent with the greater firmness (Table 1), differences in external color (Table 2), and reduction/delay in respiration and ethylene peaks (Figure 3) observed in the coated fruit, all of which are indicative of slower ripening. The gas barrier effect of the coatings, reinforced in the HS-L-LAE^®^ coating, suggests that the physiological processes responsible for the loss of texture and organoleptic quality are effectively modulated. This supports the efficacy of both formulations in slightly extending the shelf-life of avocados at 20 °C compared to the control, which would benefit marketing and consumption.

## 4. Conclusions

This work demonstrates the potential of active coatings made from avocado by-products, enriched or not with ethyl lauroyl arginate (LAE^®^), as a sustainable strategy for controlling *Colletotrichum gloeosporioides* during storage at 12 and 22 °C, as well as for extending fruit shelf life at 20 °C after cold storage at 5 °C. Coating formulations exhibited physicochemical properties suitable for industrial application, such as low viscosity, good stability and redispersibility. The incorporation of LAE^®^ significantly enhanced the coatings’ antifungal activity, particularly at 12 °C, reducing anthracnose incidence and severity. Furthermore, the coatings slightly delayed avocado ripening at 20 °C, as evidenced by the lower respiration rate, reduced ethylene production, and improved firmness and color retention. In addition, the coatings did not negatively affect sensory quality or cause physiological disorders. Further optimization of the coating formulation and application parameters may enhance performance and reproducibility under commercial conditions. Moreover, the potential incorporation of lipid-based components could be explored in future formulations to improve gas barrier properties and enable a more comprehensive modulation of fruit physiological responses, while maintaining the antimicrobial functionality of the system. These results support the use of bioactive coatings derived from avocado waste as an environmentally friendly and effective alternative to improve postharvest avocado preservation, in line with the principles of the circular economy.

## Figures and Tables

**Table 1:** Firmness (N), dry matter content (%), and weight loss (%) of ‘Hass’ avocados uncoated (control) or coated with HS-L or HS-L-LAE^®^ after storage for 2 weeks at 5 °C followed by 2, 4, 7 and 10 days at 20 °C.

Treatment	2 Weeks at 5 °C	2 Weeks 5 °C + 2 Days 20 °C	2 Weeks 5 °C + 4 Days 20 °C	2 Weeks 5 °C + 7 Days 20 °C	2 Weeks 5 °C + 10 Days 20 °C
Firmness (N)					
Control	160 ± 70 ^a^	5.0 ± 1.0 ^a^	2.07 ± 0.17 ^a^	1.8 ± 0.4 ^a^	1.8 ± 0.4 ^a^
HS-L	150 ± 70 ^a^	5.8 ± 1.6 ^a^	2.4 ± 0.2 ^b^	2.0 ± 0.5 ^a^	2.0 ± 0.5 ^ab^
HS-L-LAE^®^	170 ± 40 ^a^	6.0 ± 1.7 ^a^	2.7 ± 0.5 ^b^	2.3 ± 0.4 ^b^	2.1 ± 0.4 ^b^
Dry matter content (%)					
Control	28 ± 2 ^a^	27.6 ± 0.8 ^a^	28 ± 3 ^a^	26 ± 2 ^a^	27.7 ± 1.4 ^a^
HS-L	29.3 ± 1.6 ^a^	26.7 ± 1.6 ^a^	28 ± 3 ^a^	27 ± 2 ^a^	27.9 ± 1.8 ^a^
HS-L-LAE^®^	28.4 ± 0.6 ^a^	27.1 ± 1.0 ^a^	26.2 ± 1.2 ^a^	27.2 ± 1.0 ^a^	28 ± 3 ^a^
Weight loss (%)					
Control	0.95 ± 0.18 ^a^	1.7 ± 0.3 ^a^	2.5 ± 0.4 ^a^	3.9 ± 0.6 ^a^	5.1 ± 0.7 ^a^
HS-L	1.1 ± 0.2 ^a^	1.8 ± 0.3 ^a^	2.7 ± 0.5 ^a^	4.2 ± 0.7 ^a^	5.3 ± 0.9 ^a^
HS-L-LAE^®^	1.11 ± 0.15 ^a^	1.70 ± 0.14 ^a^	2.6 ± 0.2 ^a^	4.1 ± 0.4 ^a^	5.4 ± 0.4 ^a^

Dry matter content at harvest: 28.7 ± 1.3%. Firmness at harvest: 240 ± 20 N. Values are means ± SE. For each assay and storage period, columns with different letters are different based on Fisher’s protected LSD test (*p*< 0.05).

**Table 2:** Values of skin and flesh color parameters (CIELAB parameters) of uncoated (control) ‘Hass’ avocados and avocados coated with HS-L or HS-L-LAE^®^ after storage for 2 weeks at 5 °C followed by 2, 4, 7 and 10 days at 20 °C.

Storage	Treatment	External Color			Internal Color		
		L*	C*	Hue	L*	C*	Hue
Harvest		35 ± 2	20.2 ± 1.0	130 ± 30	81 ± 2	47 ± 2	105 ± 3
2 weeks 5 °C	Control	35 ± 3 ^b^	22 ± 4 ^a^	125 ± 3 ^b^	80 ± 3 ^a^	42 ± 4 ^ab^	107 ± 4 ^a^
	HS-L	33 ± 2 ^a^	21 ± 5 ^a^	119 ± 4 ^a^	79 ± 2 ^a^	43 ± 3 ^b^	107 ± 3 ^a^
	HS-L-LAE^®^	31.9 ± 0.6 ^a^	23 ± 4 ^a^	117 ± 2 ^a^	80 ± 3 ^a^	40 ± 3 ^a^	106 ± 4 ^a^
2 weeks 5 °C 2 days 20 °C	Control	33 ± 2 ^b^	15 ± 4 ^a^	95 ± 11 ^a^	79 ± 3 ^a^	40.0 ± 1.4 ^a^	108 ± 3 ^a^
	HS-L	32 ± 2 ^b^	15 ± 4 ^a^	106 ± 7 ^c^	79 ± 3 ^a^	41.0 ± 1.8 ^bc^	108 ± 3 ^a^
	HS-L-LAE^®^	30 ± 3 ^a^	16 ± 4 ^a^	100 ± 9 ^b^	80 ± 2 ^a^	40.6 ± 1.6 ^ab^	107 ± 3 ^a^
2 weeks 5 °C 4 days 20 °C	Control	28 ± 2 ^c^	10 ± 4 ^a^	63 ± 18 ^a^	79 ± 2 ^a^	39.3 ± 1.7 ^a^	106 ± 3 ^a^
	HS-L	27 ± 2 ^b^	9 ± 3 ^a^	70 ± 16 ^b^	80 ± 2 ^a^	39 ± 2 ^ab^	105 ± 3 ^a^
	HS-L-LAE^®^	26.4 ± 1.9 ^a^	10 ± 3 ^a^	72 ± 15 ^b^	79 ± 3 ^a^	40 ± 2 ^b^	107 ± 3 ^b^
2 weeks 5 °C 7 days 20 °C	Control	28.4 ± 1.6 ^c^	9 ± 3 ^a^	60 ± 20 ^a^	78 ± 3 ^a^	38 ± 2 ^ab^	105 ± 3 ^a^
	HS-L	26.5 ± 1.7 ^b^	8 ± 2 ^a^	62 ± 16 ^a^	76 ± 7 ^a^	38 ± 4 ^a^	104 ± 5 ^a^
	HS-L-LAE^®^	25 ± 2 ^a^	8 ± 2 ^a^	60 ± 20 ^a^	78 ± 3 ^a^	39 ± 2 ^b^	105 ± 3 ^a^
2 weeks 5 °C 10 days 20 °C	Control	27.0 ± 1.8 ^c^	6 ± 3 ^a^	63 ± 15 ^a^	75 ± 8 ^a^	37 ± 5 ^a^	103 ± 6 ^a^
	HS-L	26.2 ± 1.7 ^b^	5.7 ± 1.8 ^a^	60 ± 20 ^a^	77 ± 4 ^a^	39 ± 2 ^a^	105 ± 4 ^a^
	HS-L-LAE^®^	25.1 ± 1.8 ^a^	5.9 ± 1.8 ^a^	62 ± 18 ^a^	75 ± 7 ^a^	39 ± 4 ^a^	104 ± 5 ^a^

Values are means ± SE. For each color parameter and evaluation date, different letters indicate significant differences using Fisher’s protected LSD test (*p* < 0.05).

**Table 3:** Sensory quality attributes of uncoated (control) ‘Hass’ avocados and avocados with HS-L or HS-L-LAE^®^ after storage for 2 weeks at 5 °C followed by 2, 4, 7 and 10 days at 20 °C.

	Quality Attributes								
Treatment	External Brightness	External Color	Color Intensity	Flavor Intensity	Sweetness	Bitterness	Taste Intensity	Creaminess	Firmness
2 w 5 °C + 2 d 20 °C									
Control	5 ± 2 ^a^	4.1 ± 1.4 ^a^	5.9 ± 1.3 ^a^	6 ± 2 ^a^	4.2 ± 1.6 ^a^	3.0 ± 1.5 ^a^	6 ± 2 ^a^	6.8 ± 1.4 ^a^	4.2 ± 1.6 ^a^
HS-L	4.7 ± 1.5 ^a^	3.3 ± 1.4 ^a^	5.8 ± 1.7 ^a^	4 ± 2 ^a^	3.5 ± 1.3 ^ab^	4.1 ± 1.6 ^a^	5.7 ± 1.8 ^a^	4.7 ± 1.4 ^b^	5.6 ± 0.8 ^b^
HS-L-LAE	3.7 ± 1.4 ^b^	5.3 ± 1.2 ^b^	2.0 ± 0.5 ^b^	5 ± 2 ^a^	2.5 ± 1.8 ^b^	5.9 ± 1.7 ^b^	3.3 ± 0.7 ^b^	2.4 ± 1.0 ^c^	6.9 ± 1.3 ^c^
2 weeks at 5 °C + 4 days at 20 °C									
Control	6.0 ± 0.8 ^a^	7.0 ± 1.1 ^a^	2.9 ± 1.4 ^a^	5.1 ± 1.8 ^a^	4 ± 2 ^a^	1.1 ± 1.3 ^a^	6 ± 2 ^a^	6.2 ± 1.6 ^a^	5.5 ± 1.5 ^a^
HS-L	5.3 ± 1.3 ^a^	5.3 ± 1.3 ^b^	2.1 ± 1.2 ^a^	3.9 ± 1.6 ^ab^	5 ± 2 ^a^	0.6 ± 0.9 ^a^	6 ± 2 ^a^	6.8 ± 1.3 ^a^	5.2 ± 1.3 ^a^
HS-L-LAE	3.9 ± 1.2 ^b^	3.6 ± 1.8 ^c^	5.1 ± 1.6 ^b^	3.0 ± 1.4 ^b^	5.0 ± 1.9 ^a^	0.8 ± 0.9 ^a^	6.1 ± 1.9 ^a^	6.4 ± 1.9 ^a^	5.9 ± 1.5 ^a^
2 weeks at 5 °C + 7 days at 20 °C									
Control	4.6 ± 1.6 ^a^	7.4 ± 1.2 ^a^	5.0 ± 1.8 ^a^	4 ± 2 ^a^	5.2 ± 1.3 ^a^	0.6 ± 0.8 ^a^	4.7 ± 1.7 ^a^	7.4 ± 1.3 ^a^	3.3 ± 1.7 ^a^
HS-L	4.9 ± 1.4 ^a^	5.0 ± 1.8 ^b^	4.9 ± 1.7 ^a^	5 ± 2 ^a^	5.2 ± 1.8 ^a^	1.2 ± 1.4 ^a^	5.1 ± 1.8 ^a^	6.4 ± 1.9 ^a^	4 ± 2 ^a^
HS-L-LAE	3.8 ± 1.1 ^a^	6.9 ± 1.3 ^a^	5.5 ± 1.7 ^a^	5.2 ± 1.6 ^a^	6.3 ± 0.9 ^a^	0.7 ± 0.9 ^a^	5.9 ± 1.8 ^a^	7.2 ± 1.1 ^a^	5 ± 3 ^a^
2 weeks at 5 °C + 10 days at 20 °C									
Control	5.0 ± 1.6 ^a^	7.3 ± 1.1 ^a^	5.0 ± 1.6 ^a^	2.2 ± 1.5 ^a^	3 ± 3 ^a^	1.2 ± 1.5 ^a^	6.2 ± 1.8 ^a^	6.5 ± 1.7 ^a^	5.2 ± 1.9 ^a^
HS-L	4.2 ± 1.3 ^a^	7.5 ± 1.4 ^a^	4.8 ± 1.3 ^a^	1.8 ± 1.7 ^a^	4.3 ± 1.9 ^a^	0.6 ± 0.9 ^a^	5 ± 2 ^a^	5.7 ± 1.8 ^a^	4.9 ± 1.6 ^a^
HS-L-LAE	4.7 ± 1.7 ^a^	7.5 ± 1.3 ^a^	4.7 ± 1.7 ^a^	2.2 ± 1.9 ^a^	2 ± 2 ^a^	0.5 ± 1.2 ^a^	5 ± 2 ^a^	7.1 ± 1.4 ^a^	4 ± 2 ^a^

Values are means ± SD. For each value and evaluation date, different letters indicate significant differences using Fisher’s protected LSD test (*p* < 0.05).

## Data Availability

The original contributions presented in this study are included in the article/Supplementary Materials. Further inquiries can be directed to the corresponding authors.

## Supplementary Materials

The following supporting information can be downloaded at: https://www.mdpi.com/article/10.3390/foods15111951/s1, Table S1: Anthracnose severity scale (%) on avocado surfaces with corresponding description and visual appearance for each percentage.
