# Supplementary material for: Development of an Antifungal Edible Coating for Avocado Fruit from Avocado Residues By-Products Through a Circular Economy Approach
Source: Foods. 2026 Jun 1;15(11):1951. doi: 10.3390/foods15111951 (PMC13257014; doi:10.3390/foods15111951)
Supplement: Supplementary file 1 [file foods-15-01951-s001.zip › foods-4290508-supplementary.pdf]

SUPPLEMENTARY FILE

Development of an Antifungal Edible Coating for Avocado Fruit from Avocado Residues By-Products Through a Circular Economy Approach

Raquel Villanova-Estors 1,2, Laura Settier-Ramírez 1,3,*, Raquel Heras-Mozos 1, Gracia López-Carballo 1, María Bernardita Pérez-Gago 3, Lluís Palou 3, Pilar Hernández-Muñoz 1 and Rafael Gavara 1,2,*

1 Packaging Lab, Instituto de Agroquímica y Tecnología de Alimentos, IATA-CSIC, Av. Agustín Escardino 7, 46980 Paterna, Spain

2 Food Science, Technology and Management PhD Program, Universitat Politècnica de València, Camino de Vera, s/n, 46022 Valencia, Spain

3 Centre de Tecnologia Postcollita (CTP), Institut Valencià d'Investigacions Agràries (IVIA), 46113 Montcada, Spain

* Correspondence: laura.settier@iata.csic.es (L.S.-R.); rgavara@iata.csic.es (R.G.)

Table S1: Anthracnose severity scale (%) on avocado surfaces with corresponding description and visual appearance for each percentage

Severity percentage	Description	Visual appearance
**0%**	The fruit shows signs of damage, but there is no sporulation or fungal growth on any part of it. There is also no necrosis around the damage.	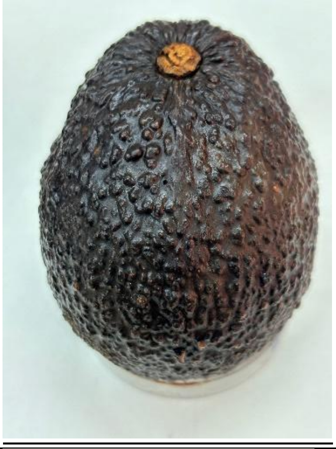
**25%**	The fruit shows fungal growth around the injury but only on one side.	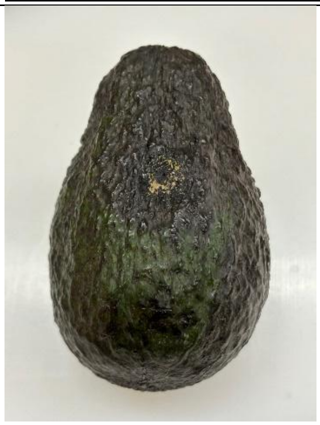

**50%**	The fruit shows fungal growth in both injuries and, in addition to sporulation, presents necrotic damage to the peel around the fungus.	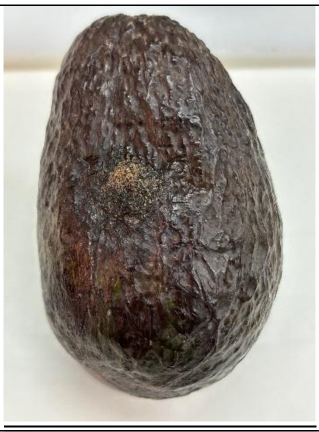
**>75%**	The fruit shows fungal growth outside the injury or a greater state of necrosis.	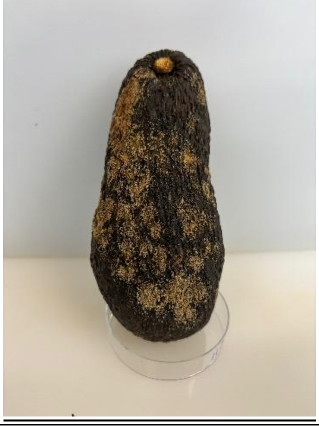
